# The Diagnostic Challenge of the Pediatric Brain Abscess

**DOI:** 10.7759/cureus.15402

**Published:** 2021-06-02

**Authors:** Kevin Rivera, Robert Truckner, Anthony Furiato, Sergio Martinez

**Affiliations:** 1 Emergency Medicine, HCA Healthcare/University of South Florida Morsani College of Medicine Graduate Medical Education Consortium: Brandon Regional Hospital, Brandon, USA; 2 Pediatric Emergency Medicine, HCA Healthcare/University of South Florida Morsani College of Medicine Graduate Medical Education Consortium: Brandon Regional Hospital, Brandon, USA

**Keywords:** altered mental status, pediatric, traumatic brain injury, increased intracranial pressure, intracranial abscess

## Abstract

Pediatric brain abscess (PBA) is a rare condition that portends a high mortality rate if not recognized and treated early. The spectrum of clinical manifestations of this disease process is wide and can often be vague, making it difficult for timely diagnosis in the emergency department. We detail the presentation of a four-year-old male with autism and a four-day history of decreased activity after a fall with a critical and rapidly worsening clinical course. Subsequent operative intervention revealed a diagnosis of PBA. This case highlights the clinical challenges of diagnosing altered mental status in a developmentally challenged pediatric patient.

## Introduction

Pediatric brain abscess (PBA) is a rare condition defined as an encapsulated area of pyogenic organisms and pus. The annual incidence of bacterial brain abscesses in the general population has been reported to be between 0.3 and 1.3 per 100,000 population; recent case series of PBAs have estimated the annual incidence to be approximately 0.5 per 100,000 children [1]. An overwhelming majority of PBAs stem from predisposing factors such as concurrent rhinosinusitis, mastoiditis, orbital cellulitis, congenital heart or lung disease, or odontogenic infection [1].

The classic symptoms and signs of brain abscesses have been reported to be headache (69%), fever (53%), and focal neurologic deficits (48%) [2]. However, in a study, the classic triad consisting of fever, headache, and focal neurologic deficits in brain abscesses was present in only 20% of patients [2]. One study found neck stiffness in 15% of patients [3]. When taking into account the insidious nature of the presentation, the vague symptomatology, and the rarity of the condition, it is no wonder that PBA is a particularly difficult diagnosis to arrive at. We report a case illustrating the vague presentation and the potential for rapid clinical decline of PBA.

## Case presentation

A four-year-old male with a history of autism spectrum disorder presented to the emergency department (ED) with a chief complaint of progressively worsening lower extremity weakness of four-hour duration. The mother reported that the patient had fallen from the bed at home approximately four days prior. She stated that the bed was slightly higher than three feet from the ground and that the patient hit his forehead. He was previously evaluated at a different ED on two separate occasions, and the mother stated that he was discharged without imaging. Over the last several days, the patient started to have a constant headache, several episodes of nonbloody, nonbilious vomiting, decreased activity, and subjective fever.

Initial vital signs were remarkable for a temperature of 99.7°F, a heart rate of 81 beats per minute, a respiratory rate of 20 breaths per minute, a blood pressure of 130/71 mmHg, and an oxygen saturation of 100% on room air. Physical examination was remarkable for a swelling to the mid-forehead without signs of basilar skull fracture. Neurological examination was remarkable for sluggish extraocular movements, somnolence, slow speech, dysmetria, and an inability to walk. The left lower extremity was found to be weak.

**Figure 1:**
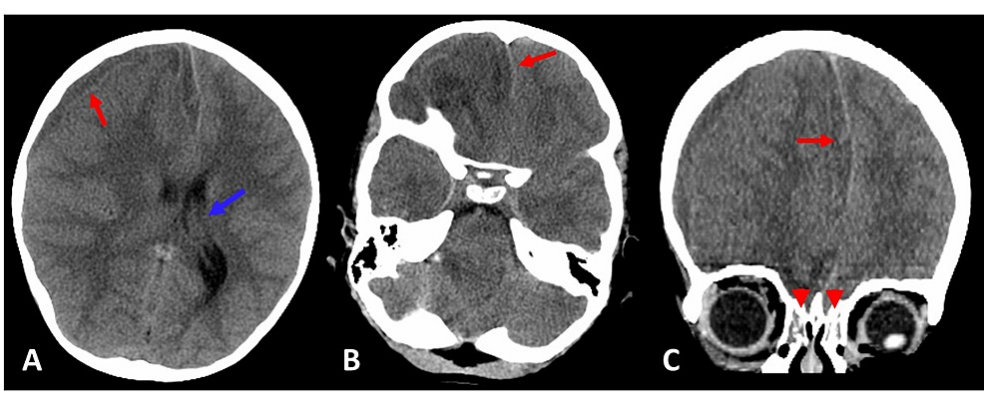
Computed tomography brain without contrast: hypodense right frontal temporal subdural hematoma extending along the anterior falx measuring 8 mm in greatest dimensions and causing right to left midline shift measuring 7 mm (red arrows). (A) Transverse view showing dilatation of the left lateral ventricle including the temporal horn concerning for mild entrapment (blue arrow). (B) More caudal transverse view showing midline shift (red arrow). (C) Coronal view showing midline shift (red arrow); there was also bilateral ethmoid sinusitis (red arrowheads).

Computed tomography (CT) imaging of the brain without contrast was performed due to suspicion of nonaccidental trauma. It showed a subdural hematoma of 8 mm in width with 7 mm of midline shift (Figure 1); ethmoiditis was also noted. Lab work was found to be remarkable for leukocytosis with monocytosis and left shift. Toxicology screenings returned presumptively positive for marijuana. A stat call was made to transfer the patient to a facility with pediatric neurosurgical services.

Glasgow Coma Score (GCS) was 14. Vital signs were within normal limits and had remained stable throughout the patient’s stay. However, a repeat temperature taken at the time of transfer showed a fever of 101.2°F. As the patient was loaded onto the ambulance, he had a seizure. He was given lorazepam and levetiracetam for seizure control with improvement and was transported emergently. Upon arrival to the accepting facility, the patient’s GCS deteriorated and he had another seizure. He was intubated and taken to the operating room emergently. Right-sided craniectomy was performed and drainage of purulent fluid was done. Blood cultures grew *Streptococcus intermedius*. The patient was placed on long-term ampicillin and showed improvement with rehabilitation.

## Discussion

PBA poses a diagnostic challenge in the ED due to its rarity, variable duration of symptoms, and nonspecific presentation. The mean duration of symptoms has been reported to be 8.3 days [2]. This patient’s clinical presentation with previous hospital visits, especially in the context of a traumatic brain injury with a low PECARN criterion and no focal neurologic deficits at the time, were also nonspecific. Per standard of care, this likely explains the previous two discharges from the hospital.

Upon arrival to our ED, the patient’s history of head trauma and a presumptive positive test for marijuana in the context of initially normal vital signs served to shift the differential diagnosis away from an infectious etiology and towards toxidromes, nonaccidental trauma, and toxic or metabolic encephalopathy. In fact, CT imaging was performed without contrast given that trauma was initially the first choice on our differential diagnosis. The patient’s suddenly deteriorating neurological status, leukocytosis, and rising temperature clarified the etiology of his symptoms later in his visit.

This initial delay in diagnosis can portend poorly to prognosis. Diagnostic delay is more likely to occur in the pediatric population [4]. This relates to the inability of pediatric patients to verbalize symptoms, especially in the context of developmental disorder, as was seen in our patient. Additionally, the classic clinical triad suggestive of brain abscess, fever, headache, and focal neurologic findings, is more specific than sensitive, as only 20% of affected patients exhibit all three at the time of diagnosis [2].

The case fatality rate has been reported to be approximately 10%, with 70% completely recovering, especially if diagnosed expeditiously [2]. The most common neurologic sequela in patients with PBA is a seizure, particularly with frontal brain abscess [5]. Poor prognostic factors for patients presenting with PBA include a rapid progression of neurologic deterioration, severe mental status changes on admission, stupor or coma (60-100% mortality), and ventricular rupture (80-100% mortality) [4].

In 40-50% of PBA cases, a contiguous site, such as the middle ear, mastoid and paranasal sinus infections, or through a skull discontinuity due to head trauma or neurosurgery, was the source of infection [6]. The most frequent site is the frontal lobe (secondary to frontal or ethmoidal sinusitis or dental infection), followed by parietal and temporal lobes (acute otitis media, mastoiditis, sphenoidal sinusitis) and less frequent sites such as cerebellum and brainstem from an otogenic or hematogenous origin [6]. A meta-analysis that included 6,663 adult and 1,023 pediatric brain abscesses reported between 1935 and 2012, in which pus or blood cultures were performed, showed that children shared similar etiology with adults, with pediatric cultures positive for *Streptococcus* spp. in 36% of cases, followed by *Staphylococcus* spp. (18%) and gram-negative enteric bacteria (*Proteus* spp., *Klebsiella pneumoniae*, *Escherichia coli*, and *Enterobacteriae*) in 16% of cases Of note, the culture growth of *S. intermedius* is consistent with the expected source of infection, given the presence of ethmoiditis on imaging [2]. The paranasal sinuses have been historically linked to streptococcal infections [7].

## Conclusions

PBA is an exceedingly rare and dangerous condition with the potential for rapid deterioration. The disease has a myriad of presentations, of which fever, headache, and neurologic deficit, as seen late in our case, are the most classic findings which may not appear together initially. The presentation of symptoms depends on the anatomical location of the abscess, microbiological composition, and vector of infection. Particularly in the setting of focal weakness or lateralizing symptoms, a high index of suspicion is required to diagnose this critical disease in a timely manner.
